# Electroporation and Electrochemotherapy in Gynecological and Breast Cancer Treatment

**DOI:** 10.3390/molecules27082476

**Published:** 2022-04-12

**Authors:** Zofia Łapińska, Urszula Szwedowicz, Anna Choromańska, Jolanta Saczko

**Affiliations:** Department of Molecular and Cellular Biology, Faculty of Pharmacy, Wroclaw Medical University, Borowska 211A, 50-556 Wroclaw, Poland; urszula.szwedowicz@student.umw.edu.pl (U.S.); anna.choromanska@umw.edu.pl (A.C.); jolanta.saczko@umw.edu.pl (J.S.)

**Keywords:** gynecological cancer, breast cancer, electrochemotherapy, electroporation, calcium electroporation

## Abstract

Gynecological carcinomas affect an increasing number of women and are associated with poor prognosis. The gold standard treatment plan is mainly based on surgical resection and subsequent chemotherapy with cisplatin, 5-fluorouracil, anthracyclines, or taxanes. Unfortunately, this treatment is becoming less effective and is associated with many side effects that negatively affect patients’ physical and mental well-being. Electroporation based on tumor exposure to electric pulses enables reduction in cytotoxic drugs dose while increasing their effectiveness. EP-based treatment methods have received more and more interest in recent years and are the subject of a large number of scientific studies. Some of them show promising therapeutic potential without using any cytotoxic drugs or molecules already present in the human body (e.g., calcium electroporation). This literature review aims to present the fundamental mechanisms responsible for the course of EP-based therapies and the current state of knowledge in the field of their application in the treatment of gynecological neoplasms.

## 1. Introduction

Despite growing awareness, screening, and intensive research into developing new therapeutic strategies, cancer remains one of the leading causes of death globally, and the number of diagnosed cases is increasing every year [1,2,3,4]. 

**Figure 1:**
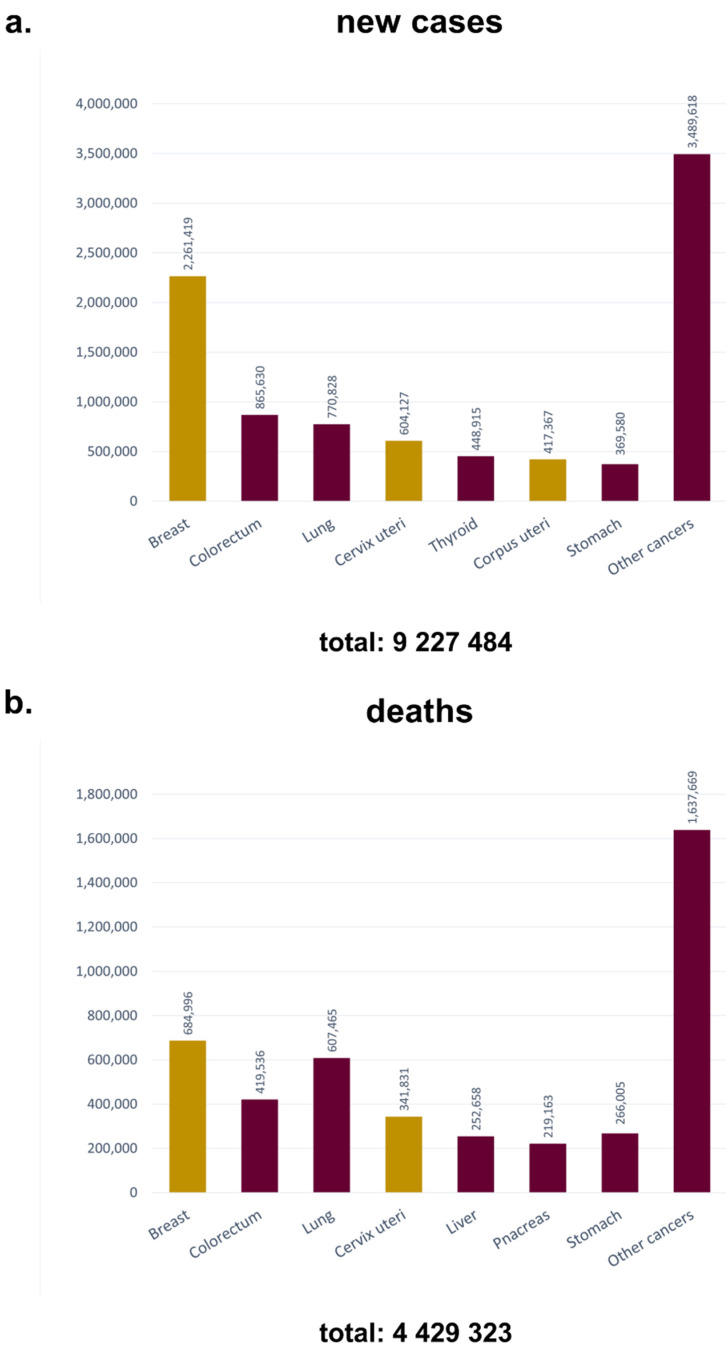
Graphs present reported Globocan 2020 data. (**a**) the number of new cases and (**b**) deaths in 2020 for women aged 0–85+ [8].

Gynecological cancers (GCs) are defined as those that originate in women’s various reproductive organs and affect women mainly in the age range of 30–75 years [5,6]. The most aggressive GCs encompass cervical, ovarian, and endometrial neoplasms [6]. Each of them is characterized by individual risk factors, epidemiology, molecular pathways, symptoms, and treatment strategies. According to the significant heterogeneity of this group of malignancies, the use of different diagnostic and treatment combinations is necessary [7]. The first-line treatment for a major group of GCs includes chemotherapy (CT) which is associated with significant side effects. The fact that a wide range of patients are diagnosed when the disease is in an advanced stage of development, and the treatment methods for progressive GCs remain limited, means that gynecological neoplasms are associated with a high mortality rate (Figure 1).

It was decided to also consider therapeutic strategies for breast cancer (BC) in the presented review. This is because, despite BC not being categorized in the GC group, it has a considerable impact on women’s lives. According to WHO (World Health Organization) statistical data, in 2020, BC had a worldwide incidence of ~2.3 million new cases and a mortality rate of nearly 700,000 deaths [1]. 

Forecasts for 2040 almost double these numbers. Therefore, considering all the issues referred to above, there is an urgent need to look for new and more effective solutions that will help eliminate, among others, the issue of multi-drug resistance of neoplasms and to reduce the number of side-effects affecting patients [4]. Hope has been provided by treatment methods based on the phenomenon of EP, which has been developing rapidly in recent years. EP-based therapies are already used to treat cutaneous and subcutaneous tumors and deep-seated tumors [9,10,11,12,13]. Their application to gynecological and breast carcinomas is still under development and requires further investigation. However, in the presented review, we have sought to summarize current knowledge and progress made.

## 2. Electroporation and Electroporation-Based Treatments in Oncology

### 2.1. The Brief Theory of Electroporation

Electroporation (EP) is a biophysical phenomenon based on the use of pulsed electric fields (PEFs) resulting in increased plasma membrane (PM) permeability. Kotnik et al. have rightly pointed out that the narrower term ‘electroporation’ and the more general ‘electropermeabilization’ are often misused as synonyms [14]. For a detailed explanation of this issue, please refer to the mentioned review. EP has found application in a wide range of disciplines, including the food industry [15], biotechnology [16,17], and medicine [12,18,19,20]. In 1982, Neumann et al. demonstrated that the use of EP to temporarily increase plasma membrane permeability enabled the transport of DNA or ordinarily non-permeable molecules, e.g., cytotoxic drugs, into the cell’s interior [21]. This paper initiated the medical application and broader study of EP. In later years, both techniques gained popularity under the names gene electrotransfer (GET) and electrochemotherapy (ECT), respectively [22,23]. These and other treatment solutions based on EP are precisely described in the following sections of the review.

Even though the exact mechanism of EP has not yet been fully elucidated, several hypotheses describing the events underlying this phenomenon have been proposed, including conformational changes of the phospholipid bilayer [24,25,26,27,28], its phase transition [29], denaturation of membrane proteins [30], and lipid oxidation affecting a wide range of PM properties [24,25,31]. However, scientists have come to a consensus that cell exposure to high-amplitude electric fields of sufficient duration results in the spontaneous formation of aqueous nanopores in PMs [14,18,32,33,34]. Furthermore, there is growing evidence that exposure to electrical impulses leads to chemical changes in lipids and membrane protein functions, resulting in increased membrane permeability [14]. 

Physiologically, every cell maintains a resting transmembrane voltage (TMV) ranging from −70 to −40 mV [14]. This term describes an electric potential difference between the inner and outer spaces of the PM, resulting from the action of the ion pumps and channels located in the PM, e.g., Na^+^ and K^+^ active and passive transport. When the cell is exposed to an external electric field, an induced transmembrane voltage (iTMV), denoted by ΔΨ_m_, occurs that is proportional to the strength of the external electric field and exists as long as the field is present [35]. 

The iTMV value for regularly shaped cells (spheroids, cylinders, etc.) with a nonconductive membrane, and which are sufficiently distant from each other, can be expressed by an explicit formula, referred to as Schwan’s equation [18]:

ΔΨ_m_ = *f ER* cos*θ* (1 − *e*^−*t*/*τ*^)

where ΔΨ_m_ is the induced transmembrane voltage, *f* is a dimensionless factor, *E* is the homogeneous electric field strength, *R* is the cell radius, *θ* is the angle measured from the center of the cell concerning the direction of the field, *t* is the time elapsed since the onset of the field, and *τ* is the time constant of membrane charging. 

**Figure 2:**
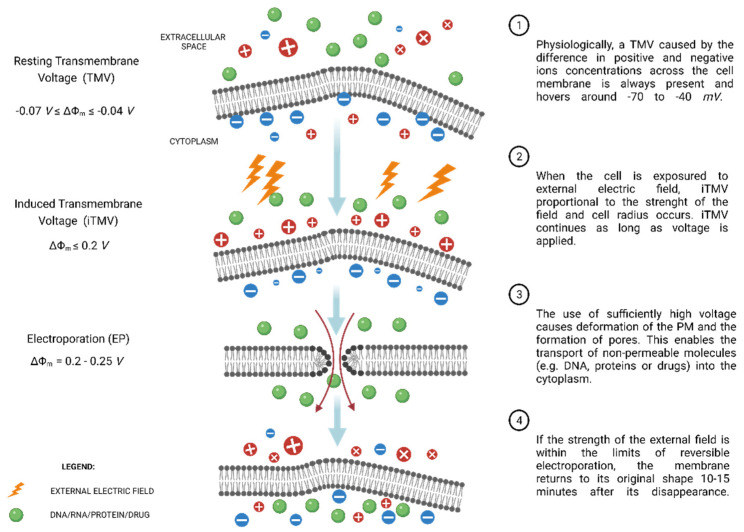
Conceptual scheme of electroporation/electrochemotherapy mechanism [14,39,40].

The application of a series of fields of adequate strength causes the occurrence of transmembrane voltages far exceeding the cell’s physiological range [14,36,37]. The effects of this event are rearrangements in the phospholipid bilayer and the formation of nanopores, as mentioned above. Ordinarily, a series of rectangular electrical pulses are applied to permeabilize the cell membrane [38]. The general scheme of the EP process is shown graphically in Figure 2.

There are two types of EP, depending on pulse duration, electric field intensity, and cell features (e.g., size, membrane curvature) [25]. Aguilar et al. refer to a “two-threshold existence”. The former is the iTMV, beyond which the phenomenon of reversible electroporation (RE) occurs. Cells can repair and close the formatted pores, reestablish metabolism over time and survive [41]. RE enables the enhanced transport of medications, gene material, small exogenous proteins, etc., without significantly affecting cell viability. The second critical iTMV is the transmembrane potential value beyond which RE becomes irreversible (IRE) [25]. It should be noted that the critical iTMV value is cell-specific. Disturbances occurring in the PM are irreversible for the cell, affecting its homeostasis and ultimately resulting in cell death. Both methods are used in clinical practice and are described more fully in the following subsections. 

Depending on the duration of the pulses, three main types of EP protocol may be distinguished: nanosecond, microsecond, and millisecond [42]. The main principle is identical for all of them and is based on the PEFs used to increase the CM permeability. However, the protocols differ in their electric field parameters, influence on targeted cells or tissues, and their range of applications in clinical settings.

Nanosecond pulsed electric field (nsPEF), also known as nanopulse stimulation, is an intensely analyzed anticancer technology [42]. This technique is based on the use of external, ultrashort (nanosecond duration), and high-voltage (kV/cm) pulses [43,44], and is characterized by low energy and non-thermal effects [45]. The primary mechanism is similar to the other PEF methods mentioned above. However, the chain of events linking the initial events occurring after nsPEF application, e.g., membrane permeabilization, with the final effects, is still not fully understood [46]. nsPEFs lead to non-stable nanoscale pores forming in the plasma membrane [47]; however, unlike other PEF methods, nanopulse stimulation can induce extra- and intracellular membrane penetration [42]. This is caused by the fact that the pulse rise time reaches full amplitude before intracellular or intraorganellar charges can redistribute it to cancel the applied field [42]. In other words, the pulse duration is shorter than the cellular membrane charging time constant [44]. The effects of this phenomenon include the following, among others: activation of signaling pathways [48,49], calcium release from the affected endoplasmic reticulum [50], dissipation of mitochondria membrane potential [51,52], cytoskeleton destruction [46,53], cell swelling and blebbing [54], and induction of cell apoptosis or necrosis [55,56,57,58]. Some articles have reported that nsPEFs induce platelet aggregation [59], and bacterial cells exposed to nsPEFs show lethal and sublethal effects [60]. 

For cells characterized by irregular shape and which are closely grouped, iTMV cannot be determined analytically, and numerical solutions must be applied [18,36,61]. An alternative involves the use of potentiometric dye [62,63]. During the application of EP-based technologies to biological tissues, their passive electric properties, such as permittivity and conductivity, should be considered [64]. Both depend on the attached electric field frequency. However, tissue permittivity is inversely proportional to the frequency value, not conductivity. Moreover, when CM reaches a state of permeabilization, its conductivity increases, due to deeper structures being electroporated by pulses of lower strength [65].

Tissues are significantly heterogeneous structures, and cells characterized by different sizes, shapes, and functions may be suspended in a more minor (e.g., epithelial tissue), or larger (e.g., bones), the volume of the extracellular matrix [64]. Moreover, tissue is surrounded by elaborate blood vessels and nerves; hence, it is difficult to anticipate their EP effects. Miklavčič et al. point out that some tissues (e.g., bone or skeletal muscle) are distinctly anisotropic; therefore, during the analysis of conductivity and permittivity values, the orientation of the electrodes relative to the tissue axis (e.g., longitudinal, transverse, or their combination) needs to be checked [64]. In fiber-organized tissues (e.g., muscles), longitudinal conductivity is significantly higher than transverse conductivity. This is because in transverse electrode orientation, the charge must overcome the extracellular matrix, which has a lower conductivity compared to cells. Moreover, tissue anisotropy is frequency-dependent, and, above a certain threshold, the anisotropic properties disappear (for muscles, in the MHz frequency range). This process, and the mechanism of tissue electrical property changes depending on illness and physiological deprivations, have been thoroughly described by Miklavčič et al. [64].

Given the circumstances described above, each aspect should be considered individually for every patient during treatment planning. Clinicians use computer simulation tools to select the appropriate electrodes, plan their placement in the targeted tissue, calculate the electric field temperature distribution, and develop the optimal protocol [66,67,68].

Technologies that enable the observation of electroporated tissue include electrical impedance tomography (EIT) [69,70] and magnetic resonance electrical impedance tomography (MREIT) [71]. Changes occurring after EP-based therapy can also be observed by computed tomography (CT) and magnetic resonance imaging (MRI) [72].

### 2.2. Irreversible Electroporation (IRE)

Irreversible electroporation (IRE) is a physical, non-thermal cancer therapy that leads to cell death via permanent membrane permeability [73] and was first proposed as a novel ablation method by Davalos et al. in 2005 [74]. As described above, the primary mechanism of IRE is based on irreversible PEF use characterized by the strength significantly exceeding a permeabilization critical threshold value [25,74]. Cells exposed to this kind of PEF are not able to restore the original plasma membrane conformation and enter the path of cell death. 

Ablation areas created by IRE are characterized by clear, well-defined boundaries, which allow precise control of the ablation zone and non-ablated tissue [75]. One of the significant advantages of IRE is that it does not require the presence of chemotherapeutic drugs [74]. This is important given the current need to reduce the number of undesirable side effects and to limit the off-target toxicity associated with conventional cancer therapies. Weaver et al. point out that IRE maintains high efficiency in tumor areas conveniently located in blood vessels that provide cooling and are not limited by the heat sink effect [38,76]. As a result, IRE is a method that effectively destroys neoplastic cells that could otherwise survive treatment with other thermal ablation methods used. Moreover, literature reports suggest that, unlike other thermal ablation technologies, IRE does not lead to the destruction of connective tissue or denaturation of collagenous and other protein and/or lipid-based structures and may be applied to the treatment of tumors localized closely to essential structures, such as bile ducts [32,77,78,79,80]. 

Zhang et al. have thoroughly summarized the local and systemic immune response mechanisms induced by IRE [81]. It has been shown that IRE causes the significant release of intracellular tumor antigens, becoming an “in situ tumor vaccine.” This observation may be used to generate an anti-tumor immune response that destroys tumor cells after ablation. This could reduce local recurrence and would also eliminate distant metastases. In light of this, Zhang et al. suggest that IRE may be regarded as a potential immunomodulatory therapy and that its combination with immunotherapy may result in synergistic effects, potentially widening the field of application of the IRE method in the clinic [81].

It has been suggested that the mechanism underlying the ability of IRE to affect only metastatic cells membranes is related to transmembrane voltage [82,83,84]. The iTMV of cancer and non-cancer cells depolarizes during proliferation, reaching a value of −15 mV. The non-cancer cell TMV value returns to −70 mV after mitotic division, in contrast to cancer cells, where the value reaches −25 mV, so the TMV is required to reach iTMV, and the critical threshold of permeabilization, is lower compared to non-cancer cells. It may be caused by the disruption of PM lipids and sterol construction and the consequential influx of sodium ions (Na^+^) into the interior of the cell with negative charge accumulation [84]. Almost a year later, Blackiston reported that modifications in chloride, sodium, potassium, and calcium channel activity also impact depolarized cancer cell TMV [85].

In recent years, the use of IRE in the clinic has increased significantly, which is reflected in the high number of clinical trials conducted using this technology (almost 53 studies with active status registered in the clinicaltrials.gov database). Moreover, the combination of IRE and immunotherapy has also been evaluated (#NCT04212026; #NCT04612530).

### 2.3. Electrochemotherapy (ECT)

The phenomenon of electrochemotherapy (ECT) was first defined by Mir et al. in 1991 [86]. ECT constitutes the intravenous or intratumoral administration of chemotherapeutic agents and the exposure of cell membranes to high-intensity, well-dosed electric pulses leading to RE [41]. The increased CM permeability enables the diffusion of low- or non-permeable drugs into the cell cytosol. Initially, ECT was intended to treat small superficial neoplastic lesions that were not amenable to surgery or radiotherapy [12,86,87]. In 2006, European guidelines for the use of ECT termed the European Standard Operating Procedures of ECT (ESOPE), and standard operating procedures (SOP), were published [88,89]. Since then, electric pulses have been delivered in a sequence of eight pulses, each 100 µs-long [90]. 

As a general rule, patients receiving ECT treatment first receive an intravenous or intratumoral administration of an anticancer drug to distribute over the targeted tissue, and then the application of the electric pulses takes place [41]. 

The main advantage of ECT is the significant reduction in chemotherapeutic dose due to the locally potentiated cytotoxic effect [41,91]. Researchers have analyzed a wide range of cytostatics, but the most satisfying results were obtained for bleomycin (BLM) and cisplatin (CSP), with toxicity increases on average of 1000- and 80-fold, respectively [86,90,92,93,94]. However, some articles have also reported the promising use of the natural compounds, doxorubicin, and 5-fluorouracil [95,96,97,98]. 

It has been shown that cells damaged after ECT release substances involving intact tumor antigen secretion [41]. Consequently, the patient’s immune system activates a tumor antigen-directed immune response, and a so-called ‘abscopal effect’ occurs, while, as a result of ECT, a systemic immune response will be triggered against distant metastases [41,99]. Other ECT effects observed include vascular disruption, hypoperfusion, decreased blood flow, and increased drug retention time due to decreased blood flow [41]. Several studies have shown that malignant cells were much more sensitive to ECT using BLM than normal cells [88,100,101]. Notably, Frandsen et al. demonstrated that ECT did not affect the size of normal fibroblast spheroids [102].

Due to the satisfying tolerance, promising results, and technological evolution of EP tools, ECT is an increasingly widely used method in oncological treatment, with new modifications being developed (e.g., calcium electroporation) [12].

### 2.4. Calcium Electroporation (CaEP)

Calcium electroporation (CaEP) is a novel modification of conventional ECT, in which chemotherapeutic treatment has been replaced by supraphysiological doses of calcium ions (Ca^2+^), as reported for the first time by Frandsen et al. in 2012 [103]. The applied EP parameters are similar to those used for ECT [104]. Solutions based on the use of non-toxic molecules are significant for clinical application, considering the side effects caused by most cytotoxic drugs (e.g., bleomycin) [102]. Moreover, EP with prior Ca^2+^ application is characterized by a long durability and lower costs [105]. CaEP’s effectiveness for the treatment of different carcinomas has already been demonstrated in in vitro and in vivo studies [102,104,105,106,107,108,109,110,111]. In combination with ECT, CaEP is currently used in more than 140 clinics in Europe as an anticancer treatment modality [112]. 

Calcium is a ubiquitous second messenger involved in many cellular processes, from transcription regulation and proliferation to cell death [102]. Under physiological conditions, there is a 10–20,000-fold Ca^2+^ concentration gradient between the extra- (10^−3^–10^−2^ M) and intracellular (10^−8^–10^−7^ M) spaces, which is strictly regulated [109,112]. Monteith et al. have precisely described aspects of the Ca^2+^-cancer signaling nexus and its role as a therapeutic target [113]. In 2012, Frandsen et al. proposed the CaEP mechanism of action [103]. In subsequent years, it has been intensively investigated, supported, and precisely described by Frandsen et al. in 2020 [112]. 

**Figure 3:**
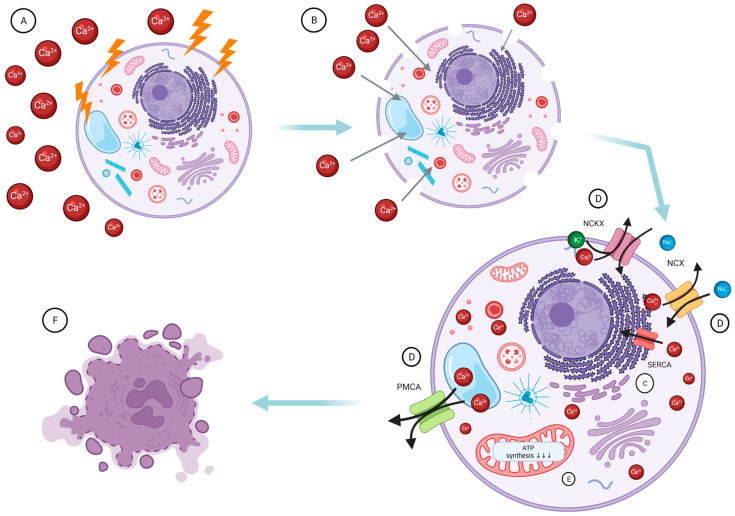
The general mechanism of calcium electroporation (CaEP). The concentration of calcium on both sides of the cell membrane (CM) is tightly controlled. The CaEP mechanism of action involves (**A**) application of Ca^2+^ supraphysiological concentration. (**B**) Subsequent application of electrical pulses which increases the permeability of the CM, which allows calcium introduction into the interior of the cell. (**C**) Ca^2+^ transportation to the mitochondria and the endoplasmic reticulum by sarco-endoplasmic reticulum calcium ATPase (SERCA). (**D**) Disruption of calcium homeostasis triggers the enhanced activity of the sodium-calcium potassium exchanger (NCKX), the sodium-calcium exchanger (NCX), and ATP-dependent plasma membrane calcium ATPase (PMCA) to extrude the extra Ca^2+^ from the cell. (**E**) At the same time, an increase in the Ca^2+^ concentration inhibits the process of ATP synthesis, leading ultimately to the complete use of its resources and cell death (**F**) [112,114].

The primary mechanism of action is based on the sudden and massive influx of Ca^2+^ following EP, resulting in intracellular calcium homeostasis disturbances, as illustrated in Figure 3 [112]. Seeking to re-establish the balance, the cell activates the ATP-dependent: Na^+^/Ca^2+^-exchanger (NCX), Na^+^/Ca^2+^/K^+^-exchanger (NCKX), and plasma membrane calcium ATPase (PMCA) to remove calcium excess from the cell (Figure 3). Simultaneously, the electrochemical gradient essential for ATP production is disturbed due to calcium overload, resulting in ATP production inhibition and ATP loss through pores created in the cell membrane [114]. Together, these events are the main cause of cell death in the wake of absolute ATP depletion, as reported in preclinical investigations [103,105,115]. Additional effects, such as lipase and protease activation and reactive oxygen species production (ROS), have also been reported [114]. 

Gibot et al. showed that, unlike ECT using BLM or CSP, CaEP does not induce genotoxicity, and its cytotoxicity is associated with ATP depletion and significant narrowing of the membrane potential [111]. Available literature sources report different types of cell death caused by CaEP therapy, depending on factors such as the cell type and morphology, time of exposure to EP, calcium concentration, etc. [112], including apoptosis, [116,117] though, necrosis is the predominant mechanism [110,115].

CaEP, as for ECT, did not affect the size of the regular spheroids and induced cell death in cancer cells more effectively [102]. Simultaneously, CaEP triggered a dramatic decrease in intracellular ATP levels in normal and malignant spheroids. Therefore, the effect of CaEP on the intracellular ATP level cannot explain the difference in cell sensitivity. However, Frandsen et al. pointed out that normal cells seem to survive this ATP depletion, whereas metabolically active malignant cells do not [103]. The differences in the vulnerability of cancer and normal cells to CaEP in other in vitro models were also previously investigated [116,117]. Notably, Frandsen et al. also indicated that normal cells could extrude the extra calcium concentration and restore it to a similar level of untreated controls approximately 4 h after treatment [110].

In 2018, Falk et al. performed the first clinical trial using CaEP to examine its effectiveness in small cutaneous metastases therapy [106]. Currently, six trials (according to the clinicaltrials.gov database, searching for “calcium electroporation”) are registered or are ongoing using CaEP, including for cutaneous and subcutaneous malignant tumors (#NCT04259658; #NCT04225767) and basal cell carcinoma (#NCT05046262). Interestingly, the use of calcium gluconate as a source of Ca^2+^ is being analyzed in a pilot study involving patients with non-curable esophageal cancer (#NCT04958044). Clinical trials involving CaEP as a therapeutical option for gynecological and breast cancer are presented in Table 1 and Table 2, respectively. 

### 2.5. Gene Electrotransfer (GET)

In recent decades, a significant evolution of gene therapies (GT) has occurred, as a result of which the treatment of hitherto non-curable diseases has become possible. GT aims at DNA or RNA transport to the targeted cell interior to genetically modify the cell by producing a protein or silencing defective or overexpressing genes [118]. This requires the genetic material to overcome several barriers, including cell membranes, nucleic barriers, etc. Therefore, the search for vectors that would facilitate this transport was initiated. The vectors which are most widely evaluated are viral vectors, employing their ability to infect and introduce gene material into the host cell [119]. Although viral vectors show satisfactory clinical efficacy, their use is associated with several drawbacks, including pre-existing immunity and the possible risk of immunotoxicity resulting from immune response activation following viral vector injection [118,120]. This situation has forced the abandonment of specific viral vectors in some countries and limits the method’s universality in using the same vector for each patient.

An alternative solution is the application of pulsed electric fields (PEFs) following naked DNA administration, termed gene electrotransfer (GET) or electrogenetherapy (EGT) [95]. Previous studies have indicated that the use of PEFs significantly enhanced gene expression compared to results obtained after naked DNA administration alone [121,122]. The GET mechanism is an elaborated and multistage process. Subsequent to PEF application, negatively charged DNA interacts with the cell membrane opposite to cathode areas [118]. PEFs, in addition to causing increased permeability of PMs, also electrophoretically push DNA towards the cell membrane. According to the available literature, the PEF strength required for DNA entrance into the cells needs to be equal to, or higher than, that required for PM electroporation [118,123,124]. In 2010, Faurie et al. indicated that DNA diffusion across the PM could take from a couple of minutes to several hours [125]. Sachdev et al., in a review, described two possible DNA entry pathways [118]. The most supported, and scientifically accepted, pathway involves the formation of DNA aggregates enclosed in the cell membrane’s vesicles. In this form, the DNA is introduced into the cell via endocytosis.

However, several defects have been highlighted for the existing method. Frequently, high voltage pulses with long duration times (millisecond pulses) are required to achieve efficient genetic material delivery [121,122,126]. However, tissue damage may occur; on the other hand, reduction in voltage inhibits transfection efficiency. The conjunction of lower voltage electrotransfer with exogenously applied heat has been reported as a solution; however, it should be noted that this method requires more specialized devices (e.g., IRE laser heating).

In 2008, the first clinical trial using GET to enhance IL-12 (interleukin 12) administration in metastatic melanoma patients was conducted by Daud et al. [22]. Almost 25 clinical trials (according to the clinicaltrials.gov database, searching “electroporation” “vaccine”) are currently being enrolled or are ongoing to evaluate the effectiveness of PEF-mediated delivery of DNA vaccines, including an HPV DNA vaccine for HPV16-positive cervical neoplasia (#NCT04131413), an HPV DNA vaccine (VGX-3100) for patients with HIV-positive high-grade anal lesions (#NCT03603808), a DNA vaccine against Puumala virus (PUUV) and Hantann virus (HTNV) (#NCT03718130), etc. Interestingly, three GET-based SARS-CoV-2 DNA vaccines are under investigation (#NCT05102643; #NCT04788459; #NCT04447781). Moreover, almost 11 active or recruiting studies were found in this database using the search terms “electroporation” or “immunotherapy”. The analyzed immunotherapies mainly concern neoplasms, including pancreatic cancer (#NCT04835402), melanoma (#NCT04526730), hepatocellular carcinoma (#NCT03630640), acute myeloid leukemia (#NCT03083054), glioblastoma (#NCT03491683), etc. 

Based on ample evidence, EP-based technologies have shown that tumors can be successfully eliminated without recurrence and with significantly lower side effects [127,128,129]. Table 1 presents the summarized merits and demerits of EP-based therapy methods described above. In the following subsections of this review, we summarize the most recent reports on the use of therapeutic methods based on the phenomenon of EP in the treatment of gynecological and breast cancer.

**Table 1:** Summarized merits and demerits of electroporation-based treatment methods.

EP-Based Method	Merits	Demerits	Ref.
**Electrochemotherapy** **(ECT)**	applied at all stages of the cell cycle enhanced cytotoxic drug transport into the cell interior lower cytotoxic drug doses introduced into an organism involving intact tumor antigen secretion	muscle contractions acute pain vascular disruption hypoperfusion decreased blood flow increased drug-retention time	[18,41,130,131]
**Irreversible electroporation** **(IRE)**	non-thermal tissue ablation applied at all stages of the cell cycle well-defined ablation area does not require chemotherapeutic drugs destroys structures not sensitive to other thermal ablation methods does not damage connective tissue, collagenous, protein, and lipid-based structures potential immunomodulatory therapy	muscle contraction and acute pain	[32,74,75,77,79,132]
**Calcium electroporation** **(CaEP)**	applied at all stages of the cell cycle does not involve cytotoxic drugs improvement of patient’s quality of life does not involve genotoxicity decreased toxic effects on normal cells	muscle contraction and acute pain	[102,111,112,130]
**Gene electrotransfer** **(GET)**	does not involve viral vectors allowing DNA macromolecule transfer	possible to apply only on a small area, surgical intervention is needed when transferring to internal organs high voltage pulses with long duration times (ms pulses) required possible tissue damage non-target specific causing some vehicle damage e.g., quantum dot aggregation	[122,124,133,134]

## 3. The Use of EP-Based Strategies in Gynecological Malignancies

### 3.1. Ovarian Cancer

Ovarian cancer (OC) is the most fatal of all female reproductive cancers, with 313, 959 new cases and 207,252 deaths reported in 2020, and a 5-year survival rate of ~48% [1,135,136]. OC rarely affects women under 30, and the risk increases with age [135]. The risk increases significantly over the age of 50. In 2019, Momenimovahed et al. reviewing studies, including age at diagnosis, observed that the median age was 50–70 years old, depending on the population [137]. OC is determined as a “silent killer” because it is diagnosed when the first symptoms appear; these symptoms can be vague (often being confused by patients for gastrointestinal complaints), and, in a large number of cases (nearly 70%), appear at advanced stages III or IV of the disease [135]. Moreover, OC, together with endometrial cancer, is characterized by poor accessibility to specimen collection, which significantly hinders the early diagnosis of these diseases [7]. Therefore, as Holcakova et al. rightly pointed out, there is an urgent need to look for new biomarkers since population screening does not exist [7].

Conventional treatment of OC involves a combination of tumor debulking surgery with the surgical staging of the affected tissue and subsequent CT [138]. The overall survival of OC patients has not improved significantly despite numerous studies focusing on this disease, progress in surgical intervention, and the development of platinum-based CT and advanced molecular-targeted therapies, such as bevacizumab and olaparib [139]. For years the medical community has had to deal with patient relapses after CT, numerous side effects, and the phenomenon of multi-drug resistance [138]. Therefore, it is necessary to constantly investigate the molecular mechanisms responsible for the development of the disease and to look for new therapeutic solutions.

The effective improvement of conventional CT (with BLM) by EP on ovarian cancer CSP-resistant cell lines (OvBH-1 and SKOV-3) was observed by Saczko et al. [138]. The authors used EP protocols ranging from 0.8 to 1.0 kV/cm × 100 µs × 1 Hz × 8 pulses preceded by suspending the cells in BLM solution prepared in EP buffer. Interestingly, Saczko et al. also compared the effectiveness of ECT with CSP to ECT using 5-fluorouracil (5-FU) on the same OC cell lines [96]. The obtained results revealed significant enhancement in the transport of both drugs after EP application. This observation is all the more intriguing as both lines are resistant to standard CSP-based CT. Resistance is the main problem in CSP-based CT and it is desirable to develop new solutions. 

Our previous article demonstrated the enhanced cytotoxic effects of CaEP (2.5 mM) against the MDAH-2774 OC cell line compared to ECT with CSP (25 µM) [140]. Three different EP protocols were used: (1) 1.3 kV/cm × 100 µs × 100 Hz × 8 pulses (ESOPE); (2) 37.5 kV/cm × 10 ns × 1 Hz × 200 pulses; and (3) 50 kV/cm × 10 ns × 1 Hz × 200 pulses. The introduction of PEFs improved the conventional CT therapeutic effect of OC cells. Moreover, the experimental results indicated lower cell viability after the µsPEF than for nsPEF. Yo-Pro-1^TM^ dye uptake analysis supported this result. 

As mentioned in the previous subsection, IRE allows for the preservation of connective tissue surrounding the tumor. This was supported by Rolong et al. who explored the use of IRE and high-frequency irreversible electroporation (H-FIRE) to induce the death of tumor-initiating cells (TICs) [141]. The paper focused on TICs, which may play a crucial role in cancer treatment failures, including ovarian cancer (OC). It has been shown that higher frequency pulses may penetrate the epithelial layer more effectively without intensive Joule heating, causing an EP effect deeper in the target tissue. Additionally, H-FIRE reduces the intensity of muscles contractions. In the presented study, scientists using mouse ovarian surface epithelial (MOSE) cells supported the cytotoxic effect of IRE on treatment-resilient cells. The researchers applied 80 monopolar, rectangular-wave pulses with 100 μs duration at a frequency of 1 Hz with 300, 375, and 450 volts. Of particular interest, was that the results obtained indicated enhanced sensitivity of MOSE tumor-initiating variants (MOSE-L_TICv_) and malignant, late-stage (MOSE-L) cells to H-FIRE compared to non-tumorigenic (MOSE-E) cells. Three different H-FIRE protocols were applied: (1) 25 cycles × 2 µs pulses × 5 µs inter-pulse delay; (2) 25 cycles × 2 µs pulses × 2 µs inter-pulse delay and (3) 50 cycles × 1 µs pulses × 2 µs inter-pulse delay. Unfortunately, it is the only published study that has focused on using H-FIRE as a potential treatment for OC. According to the authors of this review, these results are so promising that it is worth undertaking a deeper analysis of the use of H-FIRE in the treatment of ovarian cancer and other gynecological cancers. The exposure of the OC cell line (SKOV-3) to IRE was also investigated by Yao et al. [142]. Notably, the authors analyzed the effectiveness of the combination of short high-voltage (SHV: 1.6 kV × 2 μs × 1 Hz × 20 pulses) pulses with long low-voltage (LLV: 0.24–0.48 kV × 100 μs × 1 Hz × 60–80 pulses) pulses. The results showed an enhanced cytotoxic effect of SHV + LLV on SKOV-3 cells than when applied alone. The animal model’s enhanced ablation area after SHV + LLV therapy was also noted. The presented results are promising, and an analysis of larger pre-clinical models needs to be conducted. 

Interestingly, Kobayashi et al. have used EP to load tumor suppressor miRNA (miR-199a-3p) into exosomes derived from primary-cultured omental fibroblasts of OC patients and used these constructs for miRNA replacement therapy for OC patients [143]. Treatment with miR199a-3p-loaded exosomes (miR-199a-3p-Exo) suppressed c-Met expression in CaOV3, SKOV3, and OVCAR3 cell lines, thereby inhibiting cell proliferation and invasion. In experiments using xenografts, the application of miR-199a-3p-Exo significantly disturbed c-Met expression, ERK phosphorylation, and MMP2 expression in tumors. This study presents the other type of EP application in OC treatment.

Perales-Purchat et al. applied EP as an approach to deliver a designed synthetic DNA plasmid, optimized to permit high expression of an anti-HER2 (HER2—human epidermal growth factor receptor 2) antibody (HER2dMAb) and HER2 DNA-encoded bispecific T cell engagers (HER2DBiTE), into mouse anterior tibialis muscle [144]. It was reported that HER2dMAb blocked HER2 signaling, induced antibody-dependent cytotoxicity, and delayed tumor progression for HER2-expressing ovarian and breast cancer models. The HER2DBiTE was highly cytolytic and delayed cancer progression in mice. Interestingly, it was expressed in vivo for approximately four months after a single administration, allowing for frequent dose reduction, simplifying treatment techniques, and improving expression profiles.

Unfortunately, the number of clinical trials, including with patients diagnosed with OC, is very low. The only clinical trial registered in the databases clinicaltrials.gov, PubMed, and Web of Science concerned immunotherapy alone or in combination with Il-12 DNA delivered by intramuscular EP (#NCT02960594). In 2021, Ahmed-Salim reported case series, including a patient presenting with a superficial, pre-sternal mass on a background of stage III mucinous ovarian cancer, treated with CaEP for palliation [145]. The woman underwent two debulking surgeries, radiotherapy, brachytherapy, and CT. The obtained results and CT imaging revealed resolution of the lesion and that CaEP was helpful for the reduction of distressing symptoms. Details of the CaEP used are presented in Table 2.

### 3.2. Vulvar Cancer

Vulvar cancer (VC) is a rare gynecological malignancy that affects women mainly after the menopause. However, the mean age of incidence has recently fallen due to the increase in human papillomavirus (HPV) infections [146,147]. It represents 5% of all malignant neoplastic gynecological lesions. The most common subtype of vulvar malignancy is squamous cell carcinoma (SCC). Other less-common histological types of VC are melanoma, Bartholin gland adenocarcinoma, extra-mammary Paget disease, basal cell carcinoma, and verrucous carcinoma, or sarcoma [148]. There is no specific screening, and the most effective strategy to reduce VC incidence is the opportune treatment of predisposing and preneoplastic lesions associated with its development. With VC progression, most women noticed vulvar pruritus pain with a lump or ulcer. Therefore, any suspicious vulvar lesion should be biopsied to exclude a malignant lesion [146]. Two primary pathological pathways lead to vulvar SCC [149].

The first pathway is associated with keratinizing changes, which usually occur in older women and are often connected with lichen sclerosis and/or differentiated vulvar intraepithelial neoplasia. The second pathway generally occurs in younger women and is caused by infection with oncogenic strains of HPV [150,151]. Currently, lesions arising from the vulva are classified into three sub-types: low-grade squamous intraepithelial lesions (LSILs), high-grade squamous intraepithelial lesions (HSILs), and differentiated vulvar intraepithelial neoplasia (dVIN) [152]. There is no determined treatment for conditions such as lichen sclerosus. Basic measures include avoiding exposure to precipitating factors, such as local irritants, moist environments, and the use of potent topical corticosteroids [153]. dVINs represent less than 5% of preneoplastic lesions of the vulvar, but these changes show a high probability of progression to squamous vulvar carcinoma and a higher recurrence rate than HSIL. Treatment of this type of lesion of the vulva is based primarily on surgical excision with 0.5–1 cm margins [154].

Treatment of VC depends mainly on histology staging. It is predominantly surgical; however, concurrent chemoradiation is commonly used, particularly for advanced tumors. Chemoradiation is a standard procedure in locally advanced VC. It allows reducing of the lesion area and performed surgical resection in 63–92% of initially inoperable tumors [155]. Surgical management should be carried out as the most conservative operation that will cure the disease and minimize treatment-related morbidity [156,157] and negative impact on the psycho-sexual condition of patients [158,159]. A combination of radio and CT is used in women with advanced VC, in whom primary surgical resection would damage central structures (anus, urethra) [159]. It has been shown that using a combination of CSP and 5-fluorouracil is effective for preserving the anal sphincter and urethra in inoperable VC treatment [160]. Effective and safe methods for reducing vulva tumors are needed to reduce the area excised during resection of the primary lesion and in palliative cases to ensure the patient’s optimal comfort of living. Perrone et al. assessed the effectiveness of ECT in patients with primary vulvar neoplastic lesions in clinical trials. The main purpose of these studies was to determine whether ECT with BLM could effectively shrink the lesion before surgery and whether the resection area would be reduced with pre-operative ECT [161]. In studies carried out on nine patients, it was observed that there was a significant reduction in the tumor surface in eight cases, which made it possible to reduce the resection area. Consequently, four patients avoided a urethra resection, and two others a vaginal resection.

Furthermore, in six patients, the lesion shrinkage allowed excellent cosmetic repair after surgical resection, so the negative impact on the quality of the patient’s life was reduced [161]. It has also been suggested that the inflammatory infiltrate and the immune response induced by ECT improve wound healing after resection of the lesion. At the same time, chemoradiotherapy causes tissue fibrosis, which may increase the likelihood of postoperative scar necrosis and wound dehiscence. ECT was performed only once before tumor recession in the present study. Perrone et al. suggest that because neoplastic tissue is heterogeneous, not all cells are electroporated simultaneously, which results in a suboptimal therapeutic effect. Therefore, several exposures to ECT before recession may achieve a better therapeutic effect recession than after one procedure [161].

VC recurs in about 33% of cases with an approximately 70% five-year survival rate [162]. Therapeutic options are limited in cases of relapse of VC, and quality of life is poor. In 2013, Perrone et al. published the first report on palliative ECT for patients with VC who relapsed after multimodality treatments, and for whom standard therapies were unsuitable. The results were encouraging. A complete response (CR) was observed in 62.5% of cases. Relevant symptoms, such as pain, bleeding, bad smell, and urinary discomfort subsided [132]. Based on these promising results, ECT was investigated further for the palliative treatment of VC. In studies on a group of 25 patients, local control of VC was achieved in about 80% of cases, with a 48% CR rate. A total of 7 out of 25 patients underwent a second session of ECT for disease progression, achieving a 43% CR rate [131]. Similar results were observed in another clinical study [163]. Based on these findings, several clinical centers in Italy started to treat palliative patients with VC using the method. All data from the different centers were collected in a national database called ELECTRA. The obtained results indicate that ECT is currently the best palliative treatment method for patients with VC who cannot undergo surgery, have lesions resistant to chemotherapeutic agents, or have severe comorbidities. ECT treatment for VC is easy and quick to perform and has a favorable cost-effectiveness ratio. The side effects are minor, and most patients require only small doses of pain medications to treat pain associated with ECT. Moreover, unlike radiation therapy, it is possible to repeat several ETC cycles. However, more research is needed to assess the risk of BLM-induced pulmonary complications with multiple ECT sessions and to identify the best candidates for this treatment [164]. 

Ahmed-Salim et al. conducted a study involving four patients with vulval intraepithelial neoplasia (VIN) III and recurrent vulval squamous cell carcinoma treated with CaEP [145]. Details of the study are provided in Table 2.

### 3.3. Cervical Cancer

Cervical carcinoma (CC) ranks second globally in terms of the highest number of female deaths over the age of 60 [165]. The highest incidence is observed in less developed countries, especially South Africa and South America, where two to four times more cases are indicated than for breast cancer [166]. CC rarely shows symptoms in the initial stages of development but can be detected during a routine gynecological examination or cytology [167]. The first sign is lesions on the cervix, the cause of which is assessed using the Bethesda system [168]. Neoplastic changes do not always appear; the smear examination often indicates inflammation (up to 70%) and precancerous modifications. Fortunately, well-organized cytological screening and HPV triage, combined with vaccination programs in developed countries, have significantly reduced invasive cancer incidence and mortality [7].

The most common type of CC is squamous cell carcinoma, which accounts for almost 80% of all CC cases [167]. HPV is the most significant risk factor for developing cervical cancer. HPV DNA is present in over 90% of samples with a confirmed neoplastic lesion [169]. Due to the localization of the lesion during the initial stages of the disease, trachelectomy, pelvic lymphadenectomy, or radiotherapy are used as first-line treatment. Although they do not interfere with getting pregnant, all these methods significantly increase the risk of miscarriage. Therefore, other methods are sought that will not adversely affect a woman’s fertility and will be safer for those for whom CT or radiation are contraindicated [167].

Several studies have examined the effectiveness of EP-based therapies on cervical cancer cell treatment, with IRE being the most studied. Research by Qin et al. demonstrated that IRE slows the growth of both examined cell lines (HeLa and SiHa). The survival rate estimated by the CCK-8 assay decreased from ~60% to 39.69% (HeLa) and 40.71% (SiHA). CFDA-SE assay used to assess cell proliferation showed that IRE-treated cells stop dividing. The 5th and 6th generation cells were ~7.38% for the HeLa line (control ~69.77%) and for the SiHa line ~21.72% (control 86.06%). The mechanism of cell proliferation inhibition in the G1 phase is known as cell cycle arrest, preventing cells from entering other phases and dividing. This was demonstrated by a study using propidium iodide (PI) staining involving the assessment of DNA content in cell cycle analysis. According to the study, almost 59.91% of cells in both lines were stopped in the G1 phase of the cell cycle compared to the control (44.63%) [170]. Tang et al. confirmed the inhibition of cell development in this phase and a decrease in the percentage of cells in the S phase. The theory proposed is that the regulation of cyclin D1 expression and the activity of cyclin D1-CDK4 are responsible for this. The decreased expression of cyclin D1 observed in the RT-PCR study resulted in an effect on the cell cycle and a slowdown in cell proliferation [171]. In addition to the impact on the growth of HeLa and SiHa cells, the effect of applied therapy on migration, invasiveness, and cell adhesion was confirmed, which is of great importance when assessing whether IRE can increase tumor metastasis. No significant differences were observed compared to the control, with the inference that IRE neither influenced migration nor the invasiveness or adhesiveness of HeLa and SiHa cells. This may be related to the observed excellent safety of its use in vivo [170].

Other studies have focused on the parameters of EP and its various effects. Cell survival was influenced by the electric field strength, the number of pulses used, and the time taken for the measurements, in experiments by Liu et al. Staining of cells with trypan blue was performed immediately after applying EP (0.5–2.5 kV/cm) and then after 6 and 12 h. The results showed that the higher the electric field strength, the lower the cell survival rate. Importantly, staining with trypan blue showed that the highest percentage of apoptotic cells occurred for an electric field above 1 kV/cm but less than 1.75 kV/cm. Giemsa staining and immunohistochemistry results were consistent with the flow cytometry test results. IRE significantly increased cell apoptosis and caspase-3 expression, especially at an intensity above 2 kV/cm and after 24 h from the applied therapy. Slides stained with Giemsa’s reagent showed pyknosis, a characteristic of apoptosis [172].

Chai et al. investigated the effect of IRE on the whole organism using a rabbit animal model [173]. The experiment was conducted for 28 days using 90 pulses with a duration time of 70 µs and 1.5 kV/cm strength. Clinical course and histopathological examinations using hematoxylin-eosin staining first showed shedding of the mucosal epithelium and bleeding immediately after the procedure. The cervix was also swollen and painful. At the cellular level, the characteristic features of necrosis with disruption of cell membrane continuity were observed. TUNEL assay also showed significant cell apoptosis in the ablated area. In subsequent days, healing and the goal of regeneration were observed. MT staining showed increased collagen production and tissue fibrosis that increased to sizeable levels by day 28. The experiment demonstrated that the IRE treatment did not disturb the architecture of the cervix and blood vessels and nerves to such an extent that it would affect the patient’s functions and fertility in the future [173].

The use of EP to improve the administration of drugs, particularly anticancer drugs, is already widely used throughout the world. In vitro studies have investigated whether ECT would be effective in the case of cervical cancer. Yabushita et al. examined bleomycin (BLM), adriamycin, cisplatin (CSP), mitomycin C and cyclophosphamide for their efficacy in treating cervical squamous cell carcinoma. The research used the CaSki cell line. It was confirmed that EP (with a strength of 25–100 V/mm) significantly enhanced the cytotoxic effect of all analyzed drugs, especially BLM. After applying ECT with BLM (the highest analyzed concentration), cell survival decreased by almost 20%. It was also observed that using the drug before EP application gave better cytotoxic results than those obtained for BLM added after EP [10]. Ramachandran et al. used the ME180 human-cervix-derived epithelial cell line (squamous cell carcinoma) and confirmed the positive results for BLM + EP treatment. When the drug was used alone, the cytotoxic rate was ~13.4%, compared to from 18.8 to 53% for ECT using different electric pulse strengths (V/cm) [174]. 

Clinical trials involving EP for various CC types have mainly included the administration of vaccines. Trimble et al. assessed whether VGX-3100 synthetic plasmids would positively affect the progression of cervical intraepithelial neoplasia (CIN). The goal of the nearly two-year-long experiment involving 167 patients was to eliminate HPV-16 and HPV-18, the leading causes of most cervical cancers. The study confirmed that the use of EP to facilitate the delivery of the vaccine significantly increased the level of antibodies in the human body, and the results of histopathological studies confirmed a partial regression of the disease [175]. A similar experiment is currently underway with the same vaccine in a group of women with histologically confirmed high-grade squamous intraepithelial lesions (HSIL) [176].

**Table 2:** Clinical trials, preliminary studies, and case reports focusing on the use of EP-based therapies in gynecological carcinomas treatment.

Gynecological Carcinoma	Trial Type	Phase	NCT Identifier (Status)	Number of Patients	Short Description	Protocol	Study Outcome	Ref.
**Electrochemotherapy (ECT)**								
V-SCC	Prospective	II	N/A	25	ECT in elderly (median age = 85 years) patients diagnosed with V-SCC	15,000 IU/m^2^ BLM i.v. +8 pulses; 100 µs; 5 kHz 8–28 min after BLM administration	1 month after ECT: CR = 52%; PR = 28%; SD = 12%; PD = 8% 6 months after ECT SFS = 40%	[131]
V-SCC	Prospective	Preliminary study	N/A	8	safety, local efficacy, acceptability and QoL of ECT with BLM in reducing the size of tumors in patients with V-SCC	15,000 IU/m^2^ BLM i.v. +8 pulses; 100 µs; 5 kHz 8–28 min after BLM administration	CR = 62.5%; PR = 12.5%; SD= 12.5%; PD = 12.5% 50% patients alive 9 months after ECT	[132]
VC	Prospective	N/A	NCT03142061 (Completed)	50	BLM + EP of cutaneous accessible tumor tissue in patients with advanced inoperable vulva carcinoma	N/A	N/A	N/A
**Calcium Electroporation (CaEP)**								
VIN III; V-SCC; metastatic OV	Retrospective	Case report	N/A	6	CaEP in VIN and vulvar cancer	i.t. 0.5 mL/1 cm^3^ CaCl_2_ solution (10 mL of a stock 10 mmoL/10 mL solution of CaCl_2_ with 35 mL of 0.9% NaCl) +2 µs bipolar pulses; 1.3 kV/cm (520 V each polarity); 166 kHz 30 ‘trains’ of pulses	CaEP applied 10 times CR = 50%; PR = 40% For 8 episodes, symptoms improved within 6 weeks Beyond 6 weeks, symptoms eventually recurred in all patients, and 4 patients required more than one CaEP procedure.	[145]
**Gene Eectrotransfer (GET)**								
CC	Non-randomized	I, II	NCT02172911 (Completed)	10	safety and tolerability of a therapeutic DNA vaccination against HPV16 and HPV18 E6/E7 oncogenes after chemoradiation for cervical cancer	DNA-based vaccine against HPV-16/18 coinjected with an IL-12 plasmid	8/10 patients had detectable cellular or humoral immune responses against HPV antigens after chemoradiation and vaccination; 6/10 patients generated anti-HPV antibody responses 6/10 patients generated IFNγ-producing T cell responses	[177]
VC CC	N/A	II	NCT03439085	21	MEDI0457 and durvalumab for patients with recurrent/metastatic HPV-associated cancers.	7 mg IL-12/HPV DNA plasmid i.m. and via EP at W 1, 3, 7, and 12 starting W 12, cycles repeat every 8 weeks+1500 mg Durvaluma i.v. at W 4, 8, and 12 starting W 12, cycles repeat every 4 weeks up to 13 doses	21 patients were evaluated for toxicity and 19 for a response. ORR = 21%; DCR = 42%; CR = 5.3%; PR = 15.8%; SD = 21%	[178]
CC	Non-randomized	I	NCT00685412 (Completed)		evaluate the safety and tolerability of a therapeutic DNA vaccination (VGX-3100) against HPV16 and HPV18 E6/E7	i.m. injection of 3 doses 0.6/2/6 mg VGX-3100 +EP D 0, M 1 and 3	N/A	N/A
CC	Randomized	II	NCT01304524 (Completed)		a.a	i.m. injection 1 mL VGX-3100 +EP D 0, W 4 and 12	49.5% recipients and 30.6% placebo recipients (in the per-protocol analysis) 48.2% recipients and 30% placebo recipients (in the modified intention-to-treat analysis) had histopathological regression	[175,179]
CC	N/A	I	NCT01188850 (Completed)	14	evaluate the safety, tolerability, and immunogenicity of the fourth dose of Human papillomavirus (HPV) DNA plasmid (VGX-3100) + electroporation (EP) in adult females previously immunized with VGX-3100	i.m. injection of 6 mg VGX-3100 +EP in D 0	Increased immune reactivity after bosting vaccination	[180]
CC	Non-Randomized	I	NCT01634503 (Completed)	9	evaluate the safety and tolerability of DNA-based vaccine (GX-188E) administrated via EP in patients with HPV-16 or HPV-18 associated CIN III	i.m. administration of 1/2/4 mg GX-188E via EP	N/A	N/A
CC	Randomized	II	NCT02139267 (Completed)	72	a.a	i.m administration of 1 or 4 mg GX-188E via EP in W 0, 4 and 12	64 patients were included in the per-protocol analysis (V7) and 52 in extension analysis (V8) V7: 52% (33/64) V8: 67% (35/52) presented histopathologic regression after GX-188E injection 73% (V7) and 77% (V8) of the patients with histologic regression showed HPV clearance	[181]
OC	Non-randomized	I	NCT02960594 (Completed)	93	Immunotherapy alone or in combination with Il-12 DNA delivered by IM EP in solid tumors therapy	hTERT (2/8 mg) + i.m. EP +/− IL-12 (0.5/2 mg) +/− SynCon^®^ TERT (2/8 mg) D 0, W 4, 8 and 12	hTERT immunotherapy induced a de novo cellular immune response or enhanced pre-existing cellular responses to native hTERT in 96% (88/92) of patients	[182]
**Irreversible Electroporation (IRE)**								
CC	Randomized	I, II	NCT02430610 (Completed)	30	safety and efficacy of IRE for unresectable uterine cervical neoplasms	N/A	N/A	N/A

**a.a**—as above; **N/A**—not applicable; **D**—day; **W**—week; **M**—month; **mg**—milligram; **mL**—milliliter; **ms** -millisecond; **µs**—microsecond; **kV**—kilovolts; **cm**—centimeter; **i.v**.—intravenous; **i.t.**—intratumoral; **i.m.**—intramuscular; **PI**—pulse interval; **EP**—electroporation; **OR**—objective response; **CR**—complete response; **SD**—stable disease; **PD**—progressive disease; **ORR**—overall response rate; **DCR**—disease control rate; **DFS**—disease-free survival; **SAE**—serious adverse events; **SFS**—symptom-free survival; **CC**—cervical cancer; **OC**—ovarian cancer; **VC**—vulvar cancer; **V-SCC**—squamocellular vulvar cancer; **CIN III**—cervical intraepithelial neoplasia grade III; **IL12**—interleukin 12; **TIL**—tumor-infiltrating lymphocyte; **hTERT**—human telomerase reverse transcriptase.

### 3.4. Breast Cancer

Breast cancer (BC) is the most commonly diagnosed cancer and the leading cause of death among women worldwide (Figure 1) [1]. According to data collected in the GLOBOCAN database, almost 2.3 million new cases of BC were estimated in 2020; thereby, BC has surpassed lung cancer. Forecasts show that this number is expected to increase to ~3.19 million by 2040 [183]. Current treatment includes a combination of surgical resection via mastectomy or lumpectomy, irradiation, and adjuvant CT [184]. Unfortunately, these surgical solutions remain associated with significant scarring and disfigurement, which may complicate monitoring for residual tumors and recurrence of malignancy [185]. CT is not satisfactorily effective according to patient relapses, and a wide range of side effects affect patients’ quality of life [2].

Moreover, the multidrug resistance (MDR) phenomenon is a growing problem in most cases. Another difficulty associated with BC treatment is planning the appropriate therapy, taking into account histological variety, hormonal dependence, eventual overexpression of estrogen receptor α (ERα), estrogen receptor β (ERβ), human epidermal growth factor receptor 2 (HER2), and resistance against conventional treatment [186]. Therefore, there is an urgent need to develop new approaches enabling earlier detection, which will be more effective, less toxic, and associated with fewer side effects. 

Rembiałkowska et al. examined the ECT effectiveness of applying doxorubicin with EP in MCF-7/WT and MCF-7/DOX breast cancer cells sensitive and resistant to doxorubicin (DOX), respectively [95]. Interestingly, increased cytotoxicity of DOX was noted in MCF7/DOX when drug administration was combined with EP. The resistant cell line was shown to be more sensitive to electric pulses. It has been suggested that EP-based methods might be attractive for cancer treatment in human BC, especially those with developed resistance. EP enables a reduction in drug doses and exposure time in this type of cancer, diminishing the side effects of systemic therapy. Interesting changes were observed in analyzed cell lines using the electron microscope. In the case of electric pulses together with DOX, there were many differences in lysosomes. Secondary lysosomes and vacuoles with more irregular shapes were obtained from heterogeneous material. Analysis of the cellular ultrastructure showed that MCF-7/WT cells were more sensitive to electric fields and DOX than MCF-7/DOX cells. 

Due to the lack of the three main receptors, estrogen ER, progesterone, PR, and HER2, triple-negative breast cancer (TNBC) is mainly resistant to standard CT [187]. ECT might be a promising alternative method to treat TNBC. The impact of EP without anticancer drugs on MDA-MB-231 TNBC and human colon cancer (SW-480 and HCT-116) in comparison to human fibroblast cell line (MRC-5), primary human aortic smooth muscle cells (hAoSMC), and human umbilical vein endothelial cells (HUVEC) has been evaluated. The inhibition of cell proliferation after EP in a dose-dependent manner was observed. Electric pulses of strength 375–437.5 V/cm induced the IRE of cancer cells and RE of normal human cells. The lower voltage induced apoptosis as the predominant type of cell death in contrast to higher voltages, which mainly led to necrosis in human cancer cell lines. Considering the results obtained, EP might be a promising method for use in TNBC human cell lines [188]. 

Mittal et al. considered the mechanism of ECT using electrical pulses and CSP on an MDA-MB-231 cell line by quantitative proteomic analysis which correlated well with cell viability, western blot (WB), and quantitative polymerase chain reaction (qPCR) data [189]. EP with CSP was found to be involved in regulating 14 essential glycolysis proteins. EP with CSP-induced pathways also led to oxidative imbalance, increased reactive oxygen species, and apoptotic cell death. These results indicated the potential role of EP + CSP against TNBC cells. The studies were confirmed by others [190,191].

Combining calcium with EP has been tested as a new, EP-based cancer treatment modality (CaEP). In vitro study showed that an increase in supraphysiological doses of calcium ions (Ca^2+^) into cells primarily caused necrotic cell death associated with acute and critical energy depletion [102,192]. CaEP had a similar effect to ECT with anticancer drugs on breast and ovarian cancer [140,193]. Kulbacka et al. demonstrated an enhanced antiproliferative effect in MCF-7 and MCF-7/DX cells electroporated using nsPEF protocols in combination with Ca^2+^ [194]. Furthermore, it was observed that the combination of nsPEF with calcium may be used as a temporary MDR controlling tool to obtain better drug uptake. The results obtained showed that nsPEF + Ca^2+^ triggered decreased MDR1 activity, which, consequently, may disrupt cancer cells’ MDR resistance mechanism. 

IRE has also found applications in the treatment of breast cancer. Babikr et al. considered the combination of IRE alone or with Toll-like receptor (TLR)3/9 agonists (poly I:C/CpG) (IRE + pIC/CpG), PD-1 blockade (IRE +PD-1 blockade), and their combination (IRE + Combo) [195]. The results revealed effective therapeutic outcomes for IRE + Combo on two mouse breast cancer models (Tg1-1 and 4T1), suggesting that this method may represent a promising improvement for IRE ablation in cancer treatment. Zhang et al. evaluated the effects of combining IRE and photodynamic therapy (PDT) in BC cells in vitro (MCF-7 cell line) and in vivo (BALB/C mice) [196]. They reported that combining IRE and PDT enhanced anti-tumor effects in BC and that apoptosis was the primary mechanism responsible. Compared with controls, the IRE + PDT group exhibited lower levels of VEGF, CD31, TGF-β, and Ki67 indicators. Moreover, the in vivo tumor suppression rate for IRE (1200 V) + PDT (10 mg/kg) was 68.3%. 

Bazzolo et al. investigated the response of breast cancer cell (HCC1954) culture on electrospun poly (є-caprolactone) (PCL) fibrous scaffolds to ECT therapy with bleomycin (1.0 kV/cm ± 10 µM bleomycin) to evaluate it as a potential tool for the study of EP and its applications [197]. The described three-dimensional (3D) additive manufactured PCL scaffolds revealed their potential in breast reconstruction improvement [198]. Moreover, the authors analyzed the extracellular matrix production in this kind of 3D culture. They rightly pointed out that most of the anticancer drugs selected with the traditionally used two-dimensional (2D) cell cultures have been shown to be ineffective in in vivo models. This is because 2D cultures are not able to precisely mimic the cancer environment including, for example, cell-matrix interactions. The results revealed lower sensitivity of PCL-based cultures to doxorubicin and EP/bleomycin than adherent cell cultures. The authors argue that this effect may be caused by the increased level of cancer stem cells (CSCs) detected in the proposed 3D cultures. The use of electrospun PCL cultures has also been characterized by mucopolysaccharide production and enhanced CD44 expression. Of course, further studies are essential to better understand the proposed in vitro model; however, the results obtained to date are promising. 

ECT has also been shown to effectively treat BC in clinical practice. The minor side effects of the therapy and the low intraoperative duration of cure make it possible to admit patients to the hospital for only a short time. Thus, the repeated use of ECT has enabled an increase in the rate of complete remissions. In 2013, this form of treatment, which consists of a low-dose cytostatic and EP, was also included in the Working Group for Gynaecological Oncology (AGO) mammary guidelines and the German Cancer Society (DKG). The favorable cost-benefit ratio makes this method of treatment interesting in clinical practice, and, as a result, it is already being used successfully in many German hospitals.

The first clinical trial using RE combined with a chemotherapeutic drug (ECT) was conducted in 1990–1991 [199]. Since then, numerous clinical trials applying EP-based technologies have been performed to treat small tumors, such as cutaneous and subcutaneous metastases, and larger tumors (e.g., chest wall breast cancer) [200,201]. The clinical observations using ECT with BLM in patients with BC are exciting. This study showed that small tumor BC in the absence of visceral metastasis, ER positivity, and low Ki67 index had produced a complete response to ECT with BLM. Thus, ECT was effective in BC patients. Larkin et al. noted 60% regression after ECT with BLM [202].

The first clinical trial on CaEP in breast cancer noted that CaEP could be an effective and safe treatment option [106]. Another clinical study has shown that CaEP is not inferior or less effective in comparison to BLM-based ECT, especially for cutaneous metastasis of breast cancer [108]. Details relevant to clinical trial reports the use of EP-based treatment methods in BC therapy has been shown in Table 3.

To conclude, the application of ECT and CaEP appears to be a more effective and safer treatment for breast carcinoma in vitro and in vivo. ECT has found application in the treatment of breast cancer and its metastases. Palliative effects of ECT have also been demonstrated, and pain reduction has been observed in patients. 

## 4. Conclusions

Considering increasing gynecological and breast cancer incidence, there is an urgent need to look for new, more effective, and less toxic treatment modalities. Electroporation has been investigated for the last two decades. Understanding of the basic mechanisms responsible for this phenomenon has expanded the scope of its application. EP-based therapies offer opportunities for patients who would otherwise be deprived of any alternative cancer treatment. The discovery of the stimulating effect of electroporation on the effectiveness of conventional cytostatics, which was then called electrochemotherapy (ECT), has been significant for oncology. The addition of EP to CSP-based CT enabled a significant therapeutic effect even in CSP-resistant cell lines (e.g., OvBH-1 and SKOV-3) [138,140]. The results presented in our review have also indicated that ECT and CaEP could be used as palliative therapy, its use reducing the number of side effects and improving the comfort of patients’ life [132,145]. Other EP-based methods have also shown promising results to date. As a non-thermal ablation therapy, IRE has recently found application as an alternative method of cervical and breast cancer treatment. The advantage of eP-based methods lies in the fact that they do not require the application of cytostatics and are based on naturally occurring molecules in the human body (e.g., CaEP).

Furthermore, it was indicated that most of these methods (e.g., CaEP or IRE) do not affect healthy tissue/cells at a significant level. The other important advantage is the low cost of these methods. The construction of the electroporator and electrodes does not require large financial outlays, which can significantly support oncological treatment in less affluent countries [112], especially considering that, as described above, expensive cytotoxic drugs can easily be omitted or replaced with calcium chloride (CaCl_2_). According to in vitro and in vivo studies undertaken already, this will not reduce the effectiveness of the therapy. Moreover, the EP-based therapies’ immunomodulatory properties should be more precisely investigated. Babikr et al. have rightly pointed out that tools which enable IRE-induced therapeutic immunity improvement are little known and require further investigation [195].

Each of the treatment options described above has shown promising results to date, although they still require careful evaluation. A significant breakthrough in in vitro research of EP-based therapies may be the wider use of 3D cell cultures, for example, on electrospun PCL [197]. The use of this type of synthetic polymer enables scientists to mimic the tumor microenvironment more accurately in the laboratory. This will help to more precisely predict how a tested drug or treatment will perform in vivo.

Despite a variety of advantages, EP-based therapies are still associated with some side effects. Namely, electric pulses used in IRE or ECT, for example, stimulate excitable tissues and nerves, causing pain and muscle contractions [130]. Preliminary studies have shown the potential of nanosecond-range electrical pulses to overcome this problem, but these need to be studied more precisely. Future research should focus on the search for EP protocols that will enable minimizing the doses of cytotoxic drugs or achieving similar therapeutic effects while completely eliminating them. The molecular mechanisms underlying CaEP in gynecological cancers should also be explored in more detail, and EP protocols designed to reduce or eliminate muscle contractions and acute pain that currently accompany these therapies. It should also be pointed out that the number of studies focusing on the use of EP in the treatment of gynecological and breast cancers and its effects is still negligible compared to other cancers. This is an area of oncology that deserves attention.

## Figures and Tables

**Table 3:** Clinical trials and case reports focusing on the use of EP-based therapies in breast carcinoma treatment.

Type of Therapy	Trial Type	Phase	NCT Identifier (Status)	Number of Patients	Short Description	Protocol	Study Outcome	Ref.
ECT	Prospective	N/A	N/A	39	ECT for patients with cutaneous or subcutaneous metastases with palliative intent	i.v. BLM (15,000 IU/m^2^) + EP 8 pulses; 1.0 kV/cm; PD: 100 µs; 5 kHz; needle electrodes	No SEAs were observed; ORR = 66.6%	[203]
GET	N/A	I	NCT02531425 OMS-I140 (Completed)	10	IL12 plasmid (Tavo) deliver by msEP in the TNBC treatment	Tavo (0.5 mg/mL); dose ¼ tumor volume; +i.t. EP 6 pulses; 1.5 kV/cm; pulse duration: 100 ms; PI: 300 ms 6 needle electrodes; 1.0/0.5 cm diameter	enhanced antigen presentation; enhancement of CD8+ T-cell infiltration	[204]
GET + ECT	Non-Randomized Multi-Cohort	II	NCT03567720 (Recruiting)	65	IL12 plasmid (Tavo) delivered by msEP in the TNBC treatment combined with immune- and CT therapy	Tavo + i.t. EP (every 6 W) +i.v. injected Pembrolizumab (3 weekly) +/− i.v. Abraxane^®^ (4 weekly)	N/A	N/A
ECT	Randomized	N/A	N/A	38	ECT for breast cancer metastasis to the skin and subcutaneous tissue treatment	15,000 IU/m^2^ BLM i.v. +8 pulses; pulse duration: 0.1 ms; 1.0 kV/cm 5 hHz 8–28 min after BLM administration	CR = 42% and PR = 29% 12 weeks after	[205]
GET	Randomized	I	NCT03199040 (Active, not recruiting)	13	Neoantigen DNA vaccine delivered by EP in the TNBC treatment + Durvalumab (anti-PD-L1 antibody),	Vaccine 2 i.m. EP in 2 different sites, 3 M after the standard of care (D 1) and then D 29, 57, 85, 113 and 141 +/− Durvalumab 1.5 mg every 4 W, at D 85	minimal adverse events reported;	[206,207]
GET	N/A	I	NCT02348320 (Completed)	18	Polyepitope, neoantigen DNA vaccine, delivered by EP in the TNBC treatment +/− Durvalumab after completion of a standard of care therapy	Vaccine (4 mg) + i.m. EP at D 1, 29 and 57	N/A	[207,208]
CaEP + ECT	Randomized	II	NCT01941901 (Completed)	7	The comparison between CaEP and ECT in BC treatment.	i.t. CaEP (CaCl_2_; 9 mg/mL; total dose: 0.5 mL/cm^3^ tumor volume)or BLM (1000 IU/mL; total dose: 0.5 mL/cm^3^ tumor volume) + i.t. EP 8 pulses; 0.4 kV/cm; pulse duration: 0.1 ms; 5 kHz	CaEP: OR = 72% (13/18); CR = 66% (12/18); ECT: OR = 84% (16/19) CR = 68% (13/19) no significant difference between the two treatments (*p* = 0.5) ulceration, itching and exudation reported after ECT	[106]
GET	Non-Randomized	I	NCT02960594 (Completed)	93	Immunotherapy alone or in combination with Il-12 DNA delivered by IM EP in solid tumors therapy	hTERT (2/8 mg) + i.m. EP +/− IL-12 (0.5/2 mg) +/− SynCon^®^ TERT (2/8 mg) D 0, W 4, 8 and 12	hTERT immunotherapy induced a de novo cellular immune response or enhanced pre-existing cellular responses to native hTERT in 96% (88/92) of patients	[182]
GET	Non-Randomized	I	NCT02204098 (Recruiting)	56	Mammaglobin-A DNA vaccine delivered by EP for ER+, HER2-BC patients undergoing neoadjuvant endocrine therapy or CT	+/− Neoadjuvant endocrine therapy +/− mammaglobin-A DNA vaccine (4 mg) D 28, 56, and 84 +/− EP +/− Neoadjuvant CT	N/A	N/A

**N/A**—not applicable; **D**—day; **W**—week; **M**—month; **mg**—milligram; **mL**—milliliter; **ms**—millisecond; **µs**—microsecond; **kV**—kilovolts; **cm**—centimeter; **i.v.**—intravenous; **i.t**.—intratumoral; **i.m.**—intramuscular; **CT**—chemotherapy; **EP**—electroporation; **ECT**—electrochemotherapy; **CaEP**—Calcium electroporation; **GET**—gene electrotransfer; **OR**—objective response; **CR**—complete response; **PR**—partial response; **ORR**—overall response rate; **DFS**—disease-free survival; **SAE**—serious adverse events; **BC**—breast cancer; **TNBC**—triple-negative breast cancer; **IL12**—interleukin 12; **TIL**—tumor-infiltrating lymphocyte; **hTERT**—human telomerase reverse transcriptase; **BLM**—bleomycin.

## Data Availability

Data sharing not applicable. No new data were created or analyzed in this study. Data sharing is not applicable to this article.
